# Supplementary material for: Sit4 Genetically Interacts with Vps27 to Regulate Mitochondrial Function and Lifespan in Saccharomyces cerevisiae
Source: Cells. 2024 Apr 9;13(8):655. doi: 10.3390/cells13080655 (PMC11049076; doi:10.3390/cells13080655)
Supplement: Supplementary file 1 [file cells-13-00655-s001.zip › Supplementary figures.pdf]

Supplementary figures

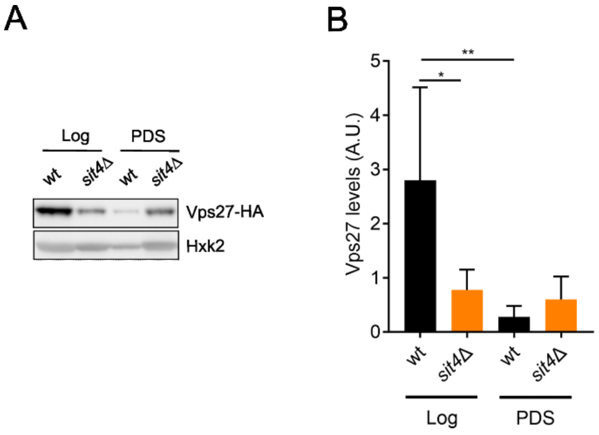

Figure S1 – *SIT4* deletion decreases Vps27 levels at the Log phase. (**A**) Cells harbouring the plasmid YCpHAC33-*Prom*-*Vps27*-*3xHA* were grown to late exponential (Log) and post-diauxic shift (PDS) phases and Vps27 levels on total protein extracts were assessed by immunodetection. Hxk2 was used as a loading control. A representative blot of at least three independent experiments is shown. (**B**) Quantification of Vps27 (normalized to Hxk2). Values are the mean ± SD; * $p \leq 0{,}05$; ** $p \leq 0.01$; one-way ANOVA.

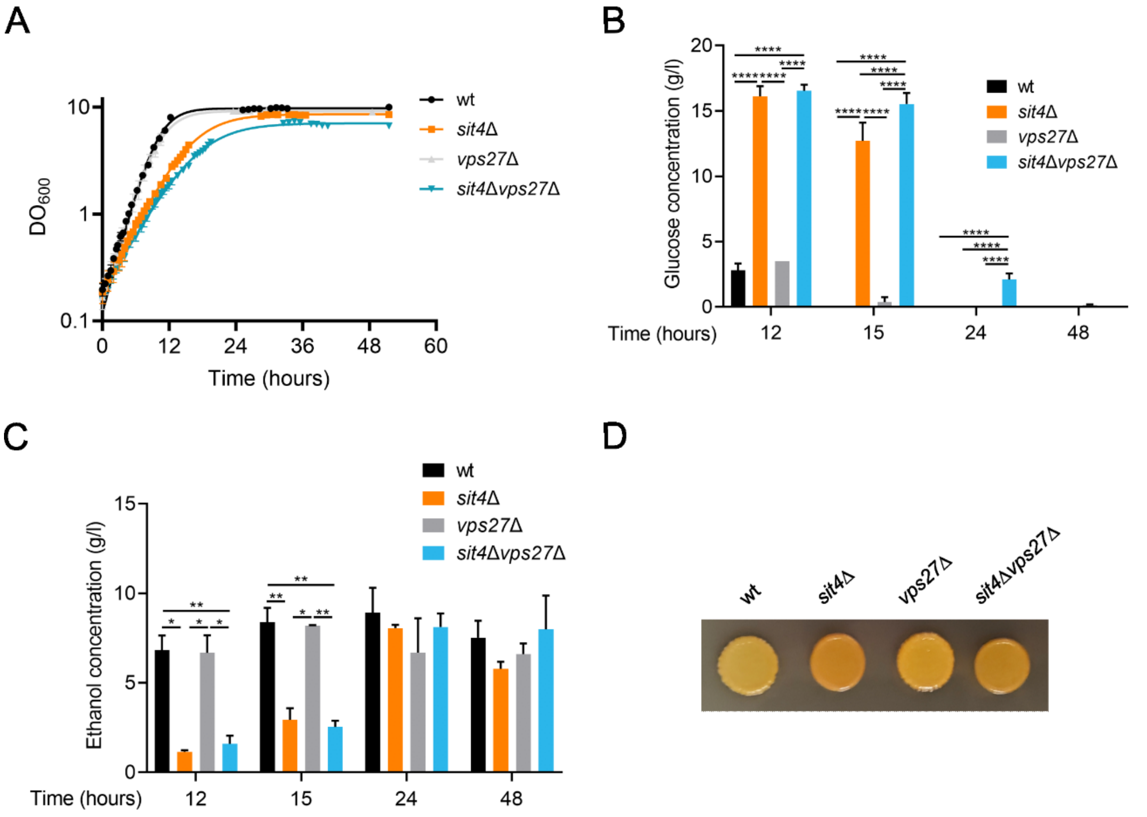

Figure S2 – Growth and metabolic profile of indicated strains. (**A**) Cells were grown until OD_{600nm}=0.2 (time 0) in SC media and the OD_{600nm} was monitored over time. Data are the mean ± SD (*n* = 2). (**B**) glucose and (**C**) ethanol levels were quantified at 12, 15, 24, and 48 h. Data are the mean ± SD (*n*≥3); * p ≤ 0,05; ** p ≤ 0.01; **** p ≤ 0.0001; one-way ANOVA. (**D**) Yeast colonies were exposed to iodine vapor to qualitatively evaluate the glycogen content. A representative figure is shown (*n* = 2).

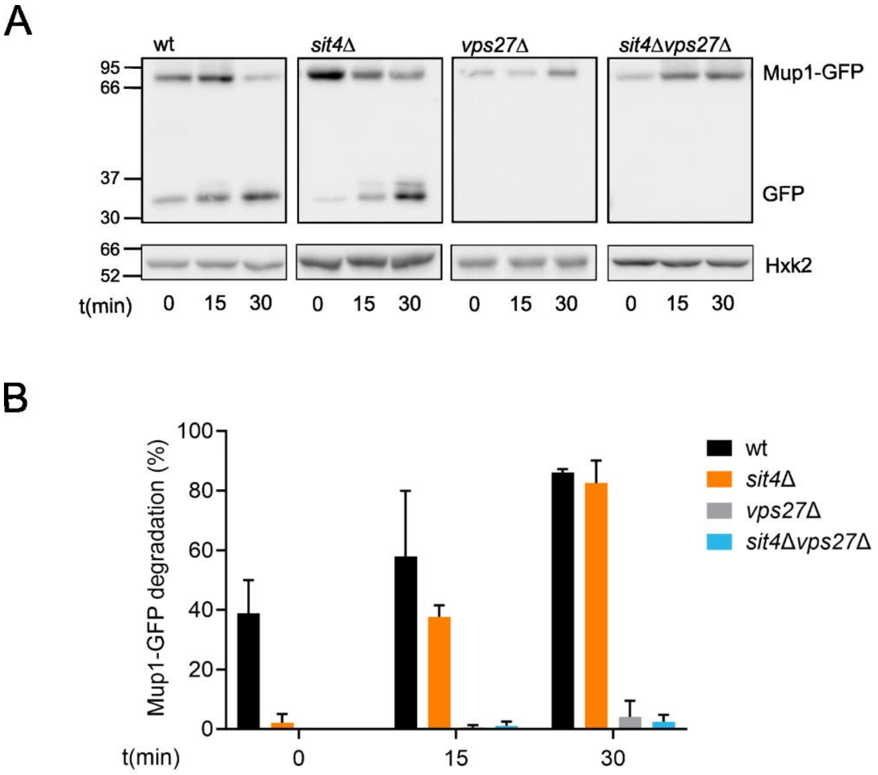

Figure S3 – Methionine increases Mup1-GFP degradation in *sit4*Δ cells. (**A**) Cells expressing pRS416-*MUP1-GFP* were grown in MM medium to the Log phase (0 min) and 38 mg L^{-1} of methionine was added as indicated. Mup1-GFP and GFP were detected by immunoblotting. Hxk2 was used as a loading control. (**B**) The induction of the MVB pathway (ratio between free GFP and the sum of GFP and Mup1-GFP) in response to methionine was evaluated. Values are the mean ± SD (*n* = 2).

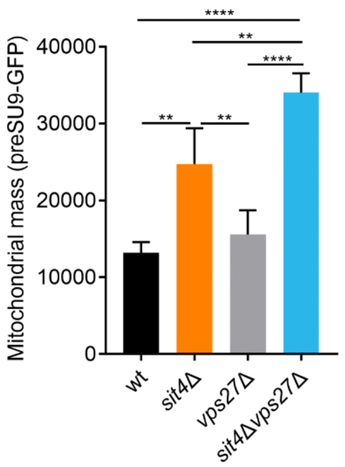

Figure S4 – Mitochondrial mass increases in *sit4*Δ and *sit4*Δ*vps27*Δ cells. Cells expressing a fusion protein containing GFP with a mitochondrial presequence (pVT100U-*preSU9*-*GFP*), were grown in SC medium to Log phase. GFP intensity was quantified by flow cytometry and used as an indicator of mitochondrial mass. Values are the mean ± SD (*n* = 4); ** p ≤ 0.01; **** p ≤ 0.0001; one-way ANOVA.

Original images

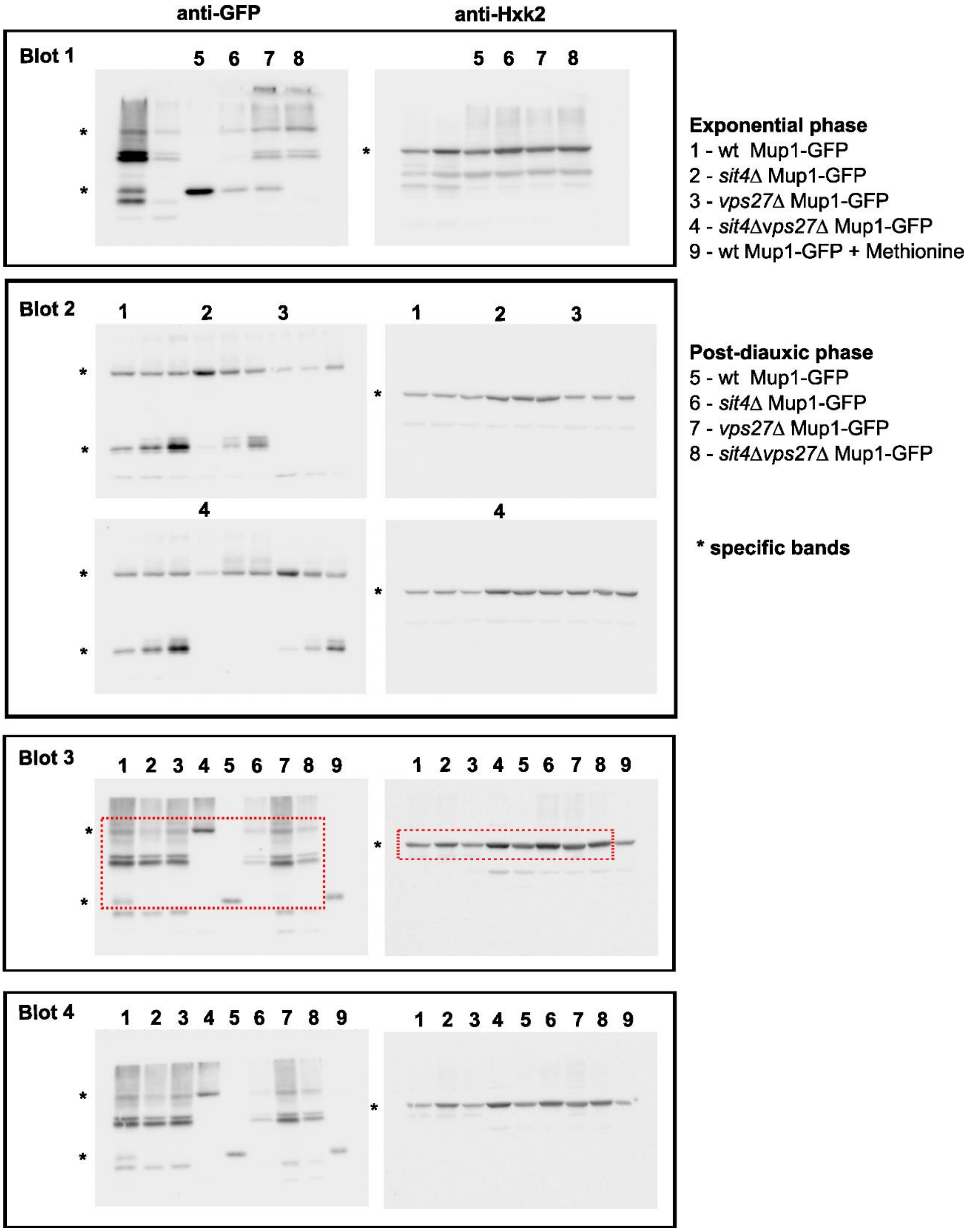

Figure S5 - Original images of Western blots used for data quantification displayed in Figures 3A and 3B. Red rectangles are used to highlight the images displayed in Figure 3A.

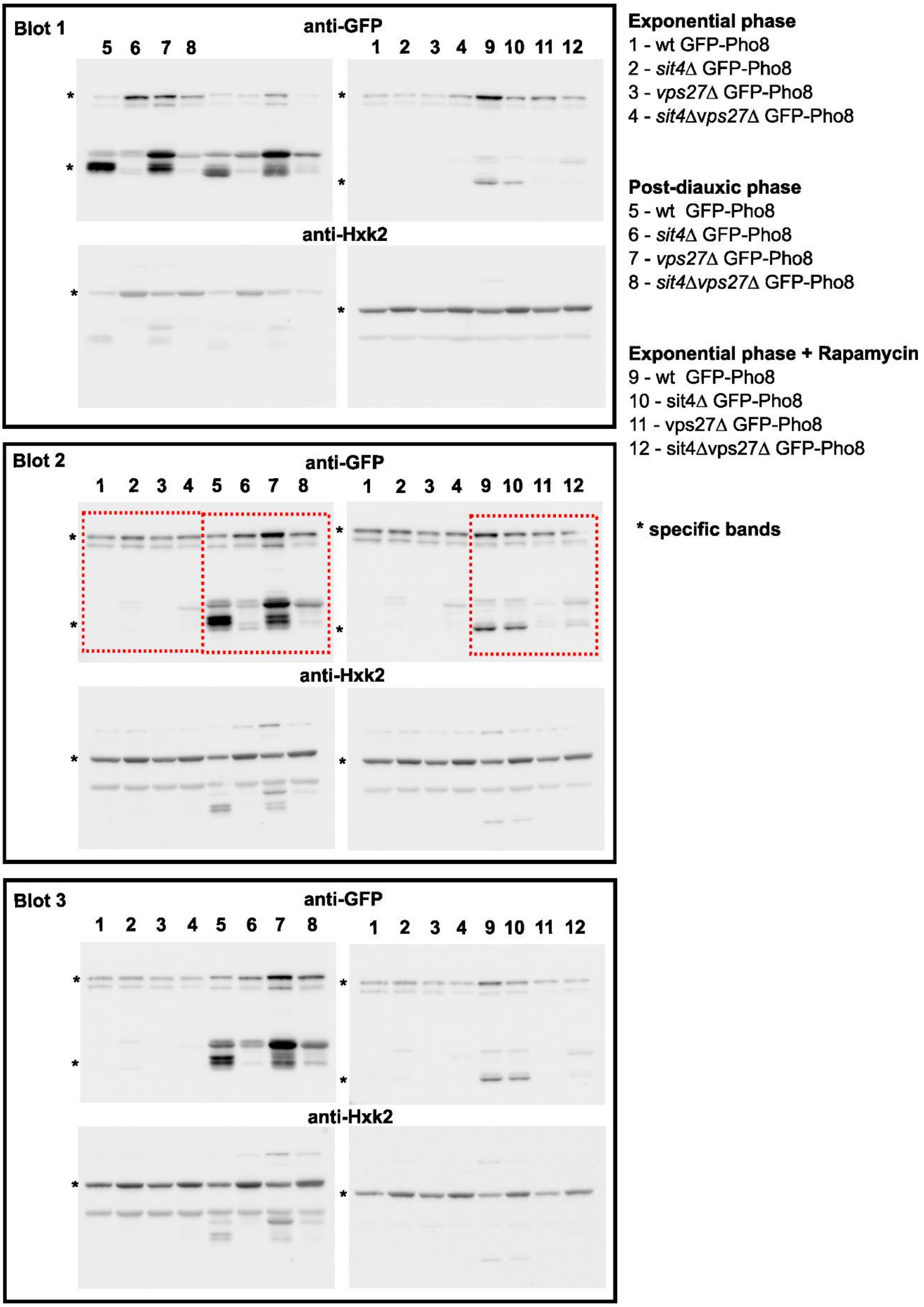

Figure S6 - Original images of Western blots used for data quantification displayed in Figures 3C and 3D. Red rectangles are used to highlight the images displayed in Figure 3C.

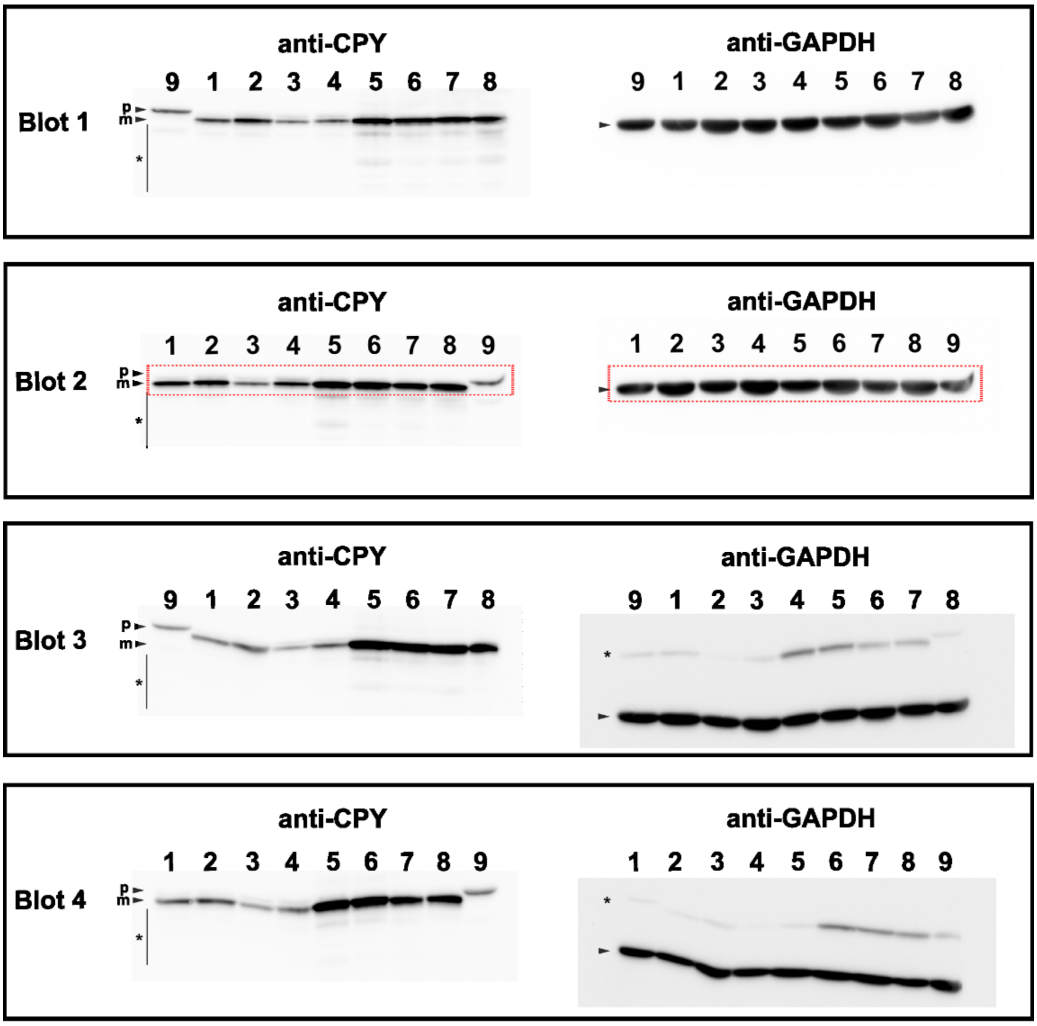

Exponential phase
1 - wt
2 - *sit4Δ*
3 - *vps27Δ*
4 - *sit4Δvps27Δ*

Post-diauxic phase
5 - wt
6 - *sit4Δ*
7 - *vps27Δ*
8 - *sit4Δvps27Δ*

Exponential phase
9 - *pep4Δ*

*** unspecific bands**

Figure S7 - Original images of Western blots used for data quantification displayed in Figures 4A and 4B. Red rectangles highlight the images displayed in Figure 4A.

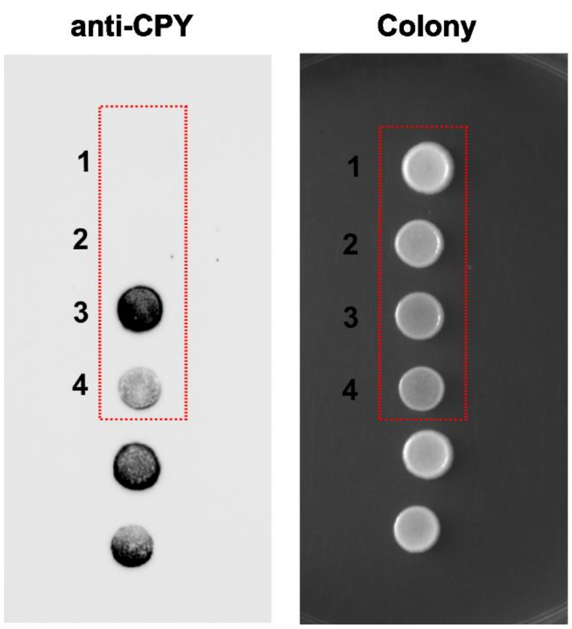

Strains
1 - wt
2 - *sit4*Δ
3 - *vps27*Δ
4 - *sit4*Δ*vps27*Δ

Figure S8 – Uncropped and unadjusted original image of the colony immunoblot displayed in Figure 4C (highlighted by the red rectangles).

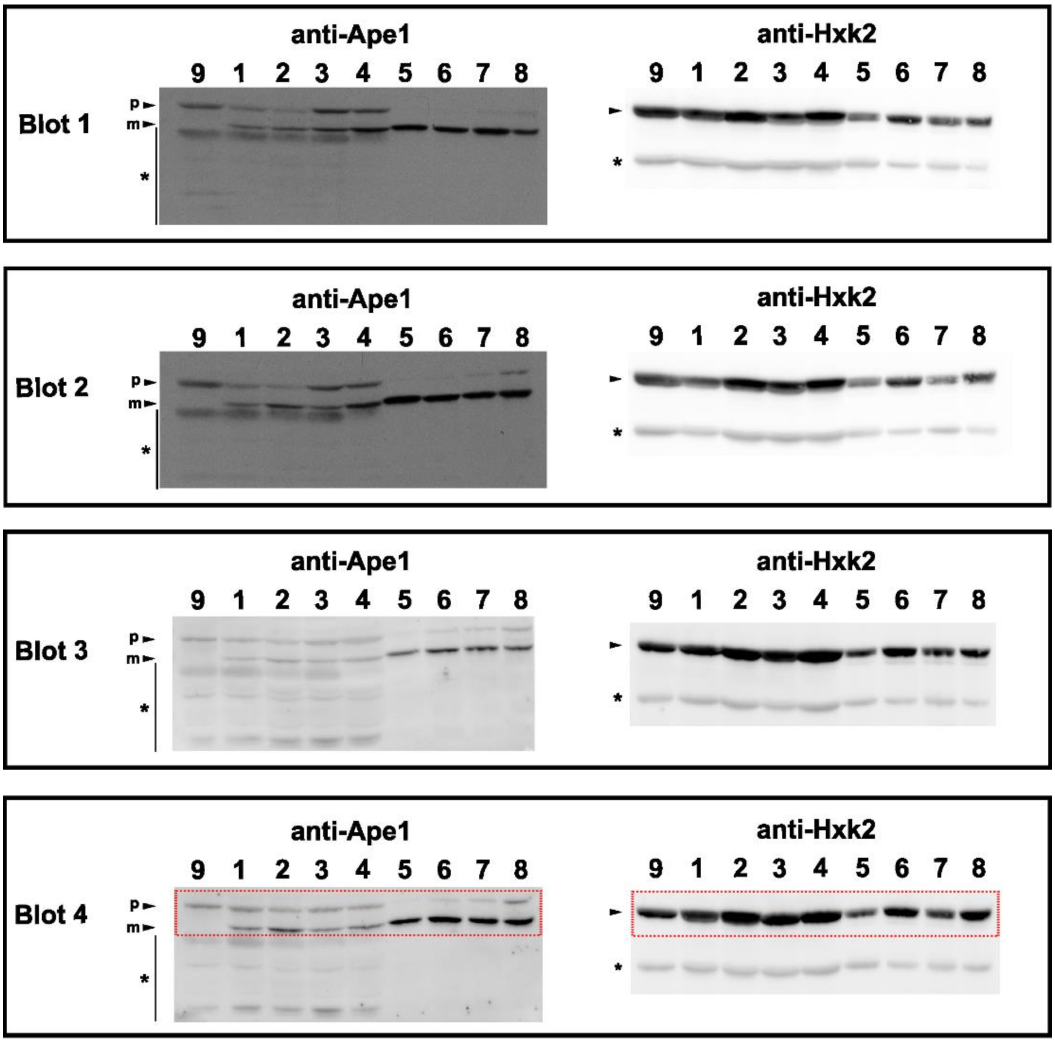

Exponential phase
1 - wt
2 - *sit4Δ*
3 - *vps27Δ*
4 - *sit4Δvps27Δ*

Post-diauxic phase
5 - wt
6 - *sit4Δ*
7 - *vps27Δ*
8 - *sit4Δvps27Δ*

Exponential phase
9 - *pep4Δ*

*** unspecific bands**

Figure S9 - Original images of the Western blots used for the data quantification displayed in Figures 5A and 5B. Red rectangles highlight the images displayed in Figure 5A.

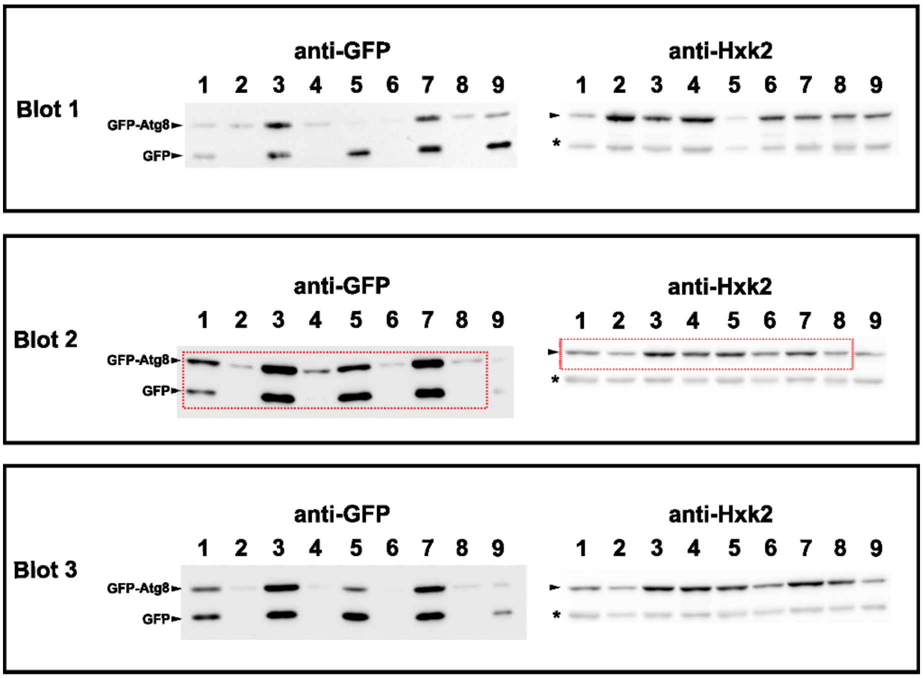

24h post-exponential phase
1 - wt + pRS416-*GFP-ATG8*
2 - *sit4*Δ + pRS416-*GFP-ATG8*
3 - *vps27*Δ + pRS416-*GFP-ATG8*
4 - *sit4*Δ*vps27*Δ + pRS416-*GFP-ATG8*

Exponential phase
9 - wt + Rapamycin

48h post-exponential phase
5 - wt + pRS416-*GFP-ATG8*
6 - *sit4*Δ + pRS416-*GFP-ATG8*
7 - *vps27*Δ + pRS416-*GFP-ATG8*
8 - *sit4*Δ*vps27*Δ + pRS416-*GFP-ATG8*

*** unspecific band**

Figure S10 - Original images of Western blots used for the data quantification displayed in Figures 5C and 5D. Red rectangles are used to highlight the images displayed in Figure 5C.

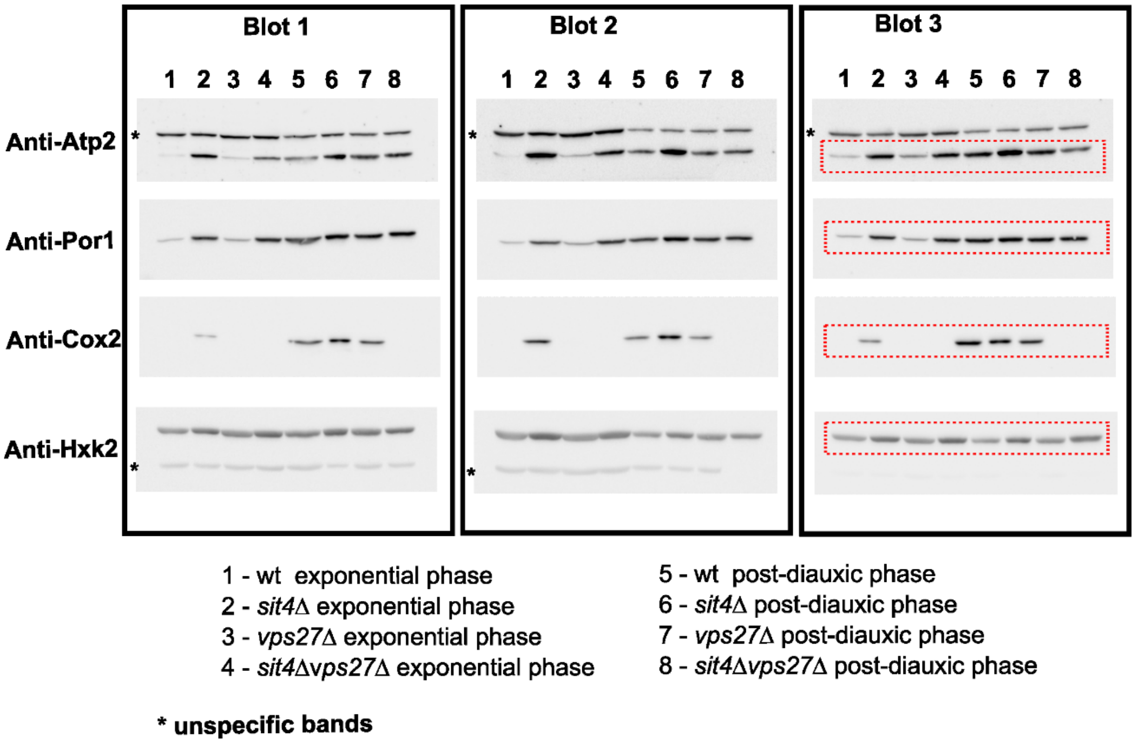

Figure S11- Original images of Western blots used for the data quantification displayed in Figures 8B-8E. Red rectangles are used to highlight the images displayed in Figures 8C-8E.

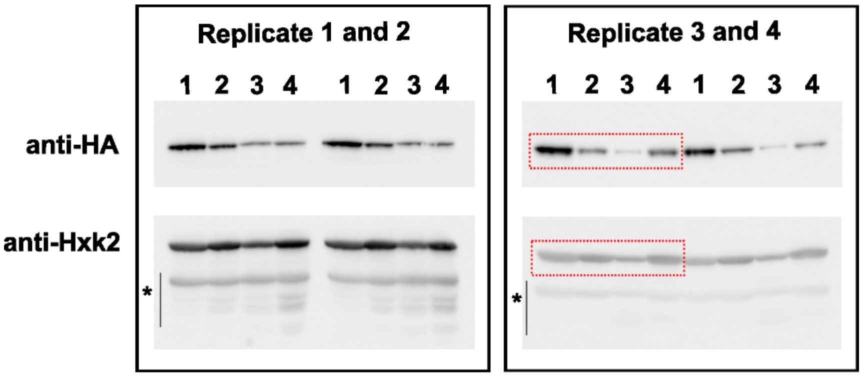

1 - *vps27Δ* Vps27-3xHA exponential phase
2 - *sit4Δvps27Δ* Vps27-3xHA exponential phase
3 - *vps27Δ* Vps27-3xHA post-diauxic phase
4 - *sit4Δvps27Δ* Vps27-3xHA post-diauxic phase

*** unspecific bands**

Figure S12 - Original images of Western blots used for the data quantification displayed in Figures S1A and S1B. Red rectangles are used to highlight the images displayed in Figure S1A.

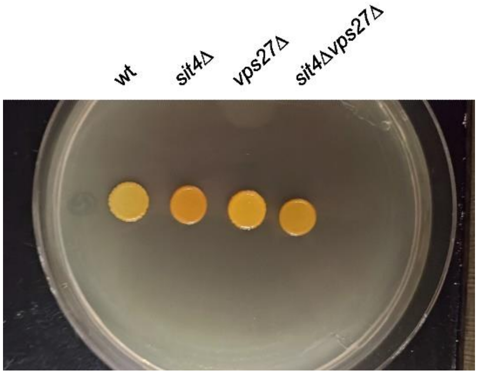

Figure S13 - Uncropped and unadjusted original image of the yeast colonies exposed to iodine vapor to qualitatively evaluate the glycogen content displayed in Figure S2D.
